# Prognosis of light chain amyloidosis: a multivariable analysis for survival prediction in patients with cardiac involvement proven by endomyocardial biopsy

**DOI:** 10.1136/openhrt-2023-002310

**Published:** 2023-07-19

**Authors:** Matthias Aurich, Julian Bucur, Johannes A Vey, Sebastian Greiner, Fabian aus dem Siepen, Ute Hegenbart, Stefan Schönland, Hugo A Katus, Norbert Frey, Derliz Mereles

**Affiliations:** 1 Department of Internal Medicine III, Cardiology, Angiology and Pneumology, University Hospital Heidelberg, Heidelberg, Germany; 2 Institute of Medical Biometry, University of Heidelberg, Heidelberg, Germany; 3 Department of Internal Medicine V, Hematology, Oncology and Rheumatology, University Hospital Heidelberg, Heidelberg, Germany

**Keywords:** Cardiomyopathy, Restrictive, Heart Failure, Systolic, Echocardiography

## Abstract

**Background:**

Cardiac involvement is a main determinant of mortality in light chain (AL) amyloidosis but data on survival of patients with cardiac AL amyloidosis proven by endomyocardial biopsy (EMB) are sparse.

**Methods:**

This study analysed clinical, laboratory, electrocardiography and echocardiographic parameters for their prognostic value in the assessment of patients with AL amyloidosis and cardiac involvement. Patients with AL amyloidosis who had their first visit to the amyloidosis centre at the University Hospital Heidelberg between 2006 and 2017 (n=1628) were filtered for cardiac involvement proven by EMB. In the final cohort, mortality-associated markers were analysed by univariate and multivariable Cox regression. Cut-off values for each parameter were calculated using the survival time.

**Results:**

One-hundred and seventy-four patients could be identified. Median overall survival time was 1.5 years and median follow-up time was 5.2 years. At the end of the investigation period, 115 patients had died. In multivariable analysis, New York Heart Association-functional class >II (HR 1.65; 95% CI 1.09 to 2.50; p=0.019), left ventricular global longitudinal strain (HR 1.12; 95% CI 1.03 to 1.22; p=0.007), left ventricular end-systolic volume (HR 1.02; 95% CI 1.01 to 1.03; p=0.001), systolic pulmonary artery pressure (HR 0.98; 95% CI 0.96 to 0.99; p=0.027), N-terminal pro-B-type natriuretic peptide (HR 1.57; 95% CI 1.17 to 2.11; p=0.003) and difference in free light chains (HR 1.30; 95% CI 1.05 to 1.62; p=0.017) were independently predictive.

**Conclusion:**

Among all patients with AL amyloidosis those with cardiac involvement represent a high-risk population with limited therapy options. Therefore, accurate risk stratification is necessary to identify cardiac amyloidosis patients with favourable prognosis. Incorporation of modern imaging techniques into existing or newly developed scoring systems is a promising option that might enable the implementation of risk-adapted therapeutic strategies.

**WHAT IS ALREADY KNOWN ON THIS TOPIC**

- Among all patients with light chain (AL) amyloidosis those with cardiac involvement represent a high-risk population with limited therapy options. Although current staging systems are powerful prognostic tools for AL amyloidosis in general, the prediction of survival in patients with cardiac involvement is not equally well investigated and patient data for endomyocardial biopsy-proven cardiac AL amyloidosis is sparse.

**WHAT THIS STUDY ADDS**

- This study could demonstrate that a relevant number of patients with cardiac amyloidosis shows long-term survival over many years. To separate patients according to prognosis, we identified different clinical, laboratory and echocardiography parameters that are independent predictors of survival in cardiac AL amyloidosis.

**HOW THIS STUDY MIGHT AFFECT RESEARCH, PRACTICE OR POLICY**

- Strain imaging holds great potential to identify cardiac amyloidosis patients with favourable prognosis as it provides direct and objective read-outs on cardiac structure and function. Modern imaging techniques might, therefore, be incorporated into existing or newly developed scoring systems to enable the implementation of risk-adapted therapeutic strategies.

## Introduction

Amyloidosis comprises a group of diseases characterised by extracellular deposition of amyloid, a fibrillar aggregation of at least 30 different misfolded precursor proteins.^1^ Light chain (AL) amyloidosis accounts for more than three-quarters of cases in patients with systemic amyloidosis and among those, another 75% suffer from cardiac involvement.^2^ With an incidence of 1.2 cases per 100 000 person-years, AL amyloidosis is a rare disease, but a trend towards higher incidence rates with time might reflect an increased awareness of the disease and diagnostic improvements.^3 4^ Nevertheless, up to 37% of patients are diagnosed more than 1 year after the onset of initial symptoms.^5^

In the case of AL amyloidosis, cardiac deposition of misfolded immunoglobulin light chain-derived amyloid fibrils disrupts tissue integrity and causes stiffening of the myocardium eventually leading to restrictive cardiomyopathy and congestive heart failure (HF).^6^ Prefibril oligomers, in addition, have direct toxic effects on cardiomyocytes and thus amplify organ dysfunction even further.^7–12^

Amyloid deposits and myocardial injury can cause elevation of cardiac biomarkers such as N-terminal pro-brain natriuretic peptide (NT-proBNP) or cardiac troponins.^13–15^ These biomarkers have accordingly been incorporated into different risk classification scores.^16–19^ While these staging systems are powerful prognostic tools for AL amyloidosis in general, the prediction of survival in patients with endomyocardial biopsy (EMB) proven cardiac involvement is not equally well investigated.

Cardiac involvement is commonly associated with a poor prognosis of AL amyloidosis. Although new therapy options have improved overall survival (OS) over the last two decades,^20^ mortality is still high among patients with confirmed or suspected cardiac involvement and after onset of HF, outcome continues to deteriorate.^21^ However, some high-risk patients survive even for years,^22 23^ raising the question whether variables not included in the established scoring systems may indicate a potentially superior therapy response and survival. Therefore, we investigated the predictive value of different clinical, laboratory, electrocardiography and echocardiograpic parameters in an initially therapy-naïve cohort of patients with AL amyloidosis and cardiac involvement proven by EMB.

## Methods

### Study design and population

The present study is a single-centre investigation based on register datasets collected at the amyloidosis centre of the University Hospital Heidelberg, Germany. The records of all patients with AL amyloidosis who had their initial visit from 27 July 2006 to 27 December 2017 were filtered for cardiac involvement that was confirmed by an EMB. In our cardiology department EMB was performed in patients referred for further invasive HF diagnostics when coronary artery disease could be excluded during heart catheterisation or in case of uncertainty of cardiac amyloidosis based on basic diagnostics (eg, imaging, biomarkers) collected in the haematology or cardiology department.

To exclude a potential bias caused by former amyloidosis treatment or by diseases other than amyloidosis that would lead to altered functional imaging parameters and elevated cardiac biomarkers, patients who were already on chemotherapy for AL amyloidosis at the time of presentation and patients with significant cardiovascular or renal comorbidities were excluded from further analysis. To ensure optimal conditions for echocardiographic measurements, in particular strain imaging, only patients in sinus rhythm and with sufficient image quality were included.

### Diagnosis of AL amyloidosis and cardiac involvement

Monoclonal gammopathy was confirmed by immunoelectrophoresis and immunofixation of serum and urine. Systemic AL amyloidosis with cardiac involvement was verified by light-microscopy immunohistochemistry and Congo red staining of myocardial biopsies showing green birefringence under polarised light.

### Clinical, laboratory, electrocardiography and echocardiography assessment

Data on demographics, medical history, physical examination, blood samples and urine samples were collected on the day of first presentation at the Heidelberg University Hospital. Additionally, a 12-lead ECG and a comprehensive echocardiography examination were performed in every patient.

Electrocardiography was analysed for heart rhythm and heart rate, cardiac axis, time intervals and low-QRS-voltage pattern (QRS complex amplitude <5 mm in any limb lead).

Echocardiography examinations were conducted using commercially available ultrasound machines (Vivid E9 BT 11, GE Vingmed Ultrasound, Horten, Norway and iE33, Philips Medical Systems, Andover, Massachusetts, USA). Three consecutive heart cycles were acquired for each two-dimensional image, with a sampling rate of 55–60 frames/s. Offline analysis was conducted according to the recommendation of the American Society of Echocardiography and the European Association of Cardiovascular Imaging^24^ using Image Arena including the 2D cardiac performance analysis software (TOMTEC Imaging Systems, Unterschleissheim, Germany).

Follow-up data were retrieved from digital clinical records and/or telephone interviews with the patients, their relatives or their family doctors.

### Statistical analysis

The study cohort was described using summary measures of the empirical distribution. Nominal variables are reported as absolute and relative frequencies and continuous variables are given as medians with 25% and 75% quantiles. In the comparison of survivors and nonsurvivors, for nominal variables, the χ^2^-test was used, and for continuous variables, the t-test for independent groups or the Mann-Whitney-Wilcoxon-test was used, as appropriate.

OS was investigated using the Kaplan-Meier estimator and the median follow-up was calculated with the reverse Kaplan-Meier method. The variables’ association with OS was examined by calculating HRs applying univariate Cox regression models, whereby NT-proBNP, high sensitivity Troponin T (hsTnT) and difference of free light chains (dFLC) were log-transformed due to their highly skewed distributions. For multivariable Cox regression modelling, the missing values (occurring in total in 1.82% of the investigated variables) were multiple (n=50) imputed. On each imputed dataset, forward selection using Cox regression was performed. In each step, the coefficients were pooled using Rubins’s rules and variable selection was done by using a criterion of p<0.1 for the pooled regression coefficients.^25^

Optimal cut-off values for risk stratification were calculated by optimising the log-rank test statistic and a 95% bootstrap CI was computed as certainty measure.^26^

Since this was a retrospective data analysis, all p values should be interpreted in a descriptive manner; p values<0.05 were accepted as significant. All statistical analyses were performed using the software R V.≥4.10.2.

## Results

**Figure 1:**
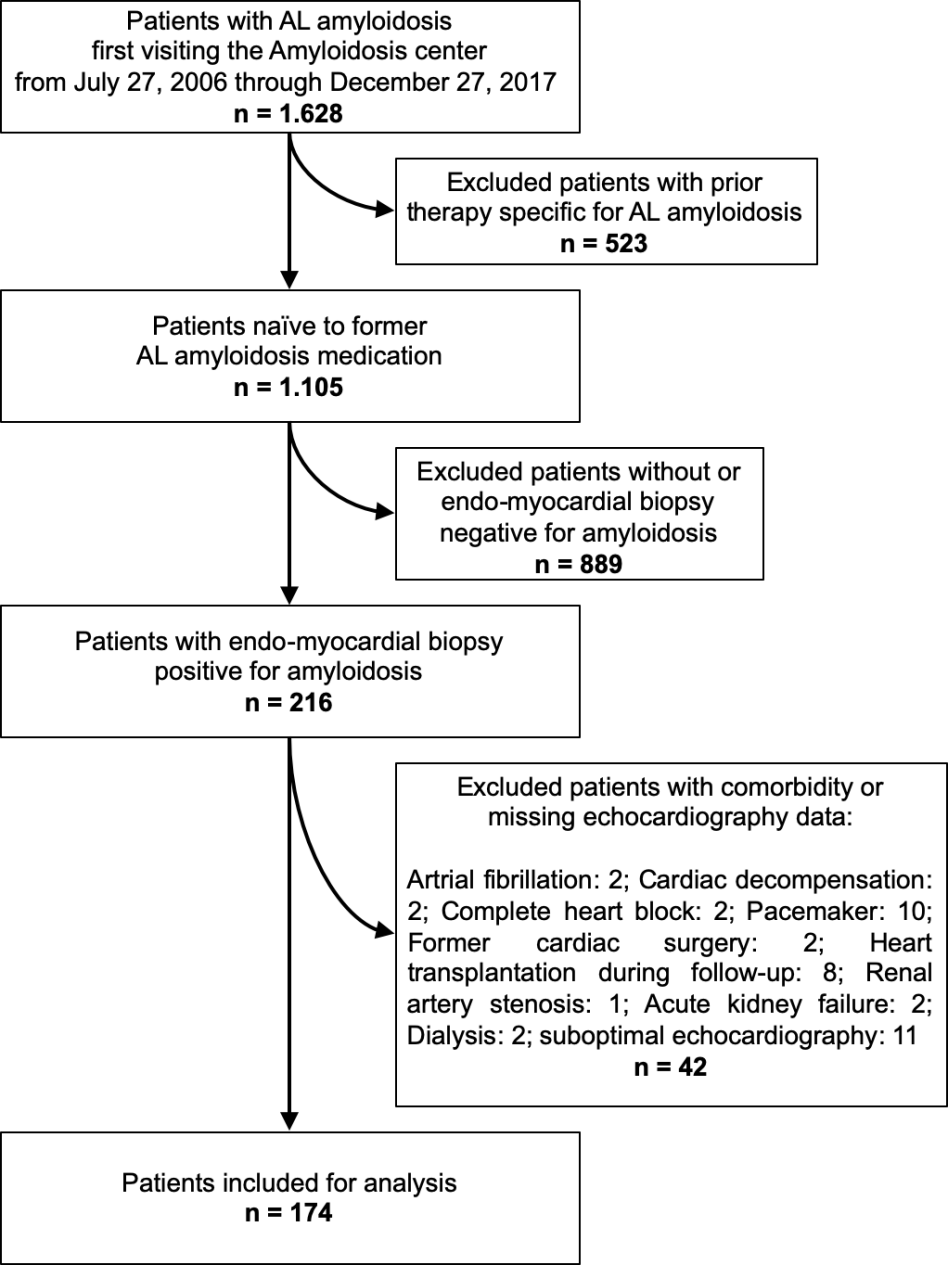
Flow chart of the inclusion and exclusion criteria of the study. AL, amyloid light chain.

From 27 July 2006 to 27 December 2017, 1628 patients with AL amyloidosis had initial contact with the amyloidosis centre at the University Hospital Heidelberg. Figure 1 depicts a flow chart of the inclusion and exclusion criteria for this study. Only patients who were chemotherapy naïve for AL amyloidosis were included. Cardiac involvement was confirmed by EMB in 216 cases. To minimise the impact of confounders, patients with known cardiovascular (atrial fibrillation n=2, cardiac decompensation n=2, complete heart block n=2, pacemaker n=10, former heart surgery n=2, heart transplantation during follow-up n=8) or severe kidney disease (renal artery stenosis n=1, acute kidney failure n=2, dialysis n=2) as well as patients with suboptimal echocardiography image quality (n=11), were excluded. The final study population consisted of 174 patients. At the end of the follow-up period in August 2021, 115 patients had died.

### Characteristics of the study population

The clinical, laboratory, electrocardiography and echocardiograpic parameters of the entire study cohort, of survivors and non-survivors are summarised in table 1. Male subjects predominated. Survivors had higher systolic blood pressure and estimated glomerular filtration rate (eGFR) whereas New York Heart Association functional class (NYHA-FC), serum levels of dFLC, NT-proBNP, hsTnT, left ventricular (LV) global longitudinal strain and the ratio of E/e’ were lower. Furthermore, pericardial effusion was less often detected in survivors. Online supplemental table 1 compares patients who survived at least 1 year with patients who died within 1 year after initial presentation, excluding censored cases. Cardiac staging according to the MAYO 3b^18^ score is presented in table 2. Among survivors most patients were in stages II and III and among non-survivors most patients were in stages III and IIIb. None of the patients met the criteria for stage I.

10.1136/openhrt-2023-002310.supp1 Supplementary data

**Table 1:** Characteristics of the study population

Parameter	Survivors (n=59)	Non-survivors (n=115)	Total (n=174)	P value
Basic and clinical data				
Male sex, n (%)	32 (54)	81 (70)	113 (65)	0.034
Age, years	62 (53; 68)	63 (56; 70)	63 (55; 69)	0.369
Hight, cm	171(165; 183)	176 (168; 181)	175 (166; 182)	0.344
Weight, kg	78 (65; 86)	77 (66; 84)	77 (66; 85)	0.963
Systolic blood pressure, mm Hg	110 (105; 125)	110 (110; 120)	110 (110; 120)	0.030
Diastolic blood pressure, mm Hg	75 (70; 80)	70 (65; 80)	70 (65; 80)	0.080
NYHA-FC>II, n (%)	26 (44)	76 (66)	102 (59)	0.005
Clinical chemistry				
Light chain type Lambda, n (%)	53 (90)	96 (83)	149 (86)	0.258
dFLC, mg/L	274 (141; 415)	325 (155; 711)	300 (152; 589)	0.033
NT-proBNP, ng/L	4528 (2472; 8482)	7516 (5046; 13 419)	6894 (3333; 11 119)	<0.001
hsTnT, pg/mL	57 (40; 89)	86 (48; 138)	71 (43; 126)	0.010
eGFR, mL/min/1.73 m^2^	81 (72;93)	74 (62;93)	77 (64;93)	0.036
ECG				
Sinus rhythm, n (%)	56 (100)	115 (100)	174 (100)	–
Heart rate, s^-1^	78 (69; 90)	82 (74; 91)	81 (71; 90)	0.452
Heart axis				0.871
Normal heart axis, n (%)	22 (37)	39 (34)	61 (35)	
Left axis deviation, n (%)	26 (44)	55 (48)	81 (47)	
Right axis deviation, n (%)	11 (19)	20 (18)	31 (18)	
PQ interval, ms	186 (166; 216)	178 (158; 208)	180 (160; 210)	0.348
QRS interval, ms	96 (88; 112)	100 (90; 112)	98 (90; 112)	0.792
QTc interval, ms	441 (421; 457)	437 (419; 456)	439 (419; 456)	0.656
Low-QRS-voltage pattern, n (%)	20 (34)	53 (47)	73 (42)	0.101
Echocardiogram				
LV-Septum, mm	17 (15; 20)	17 (15; 20)	17 (15; 20)	0.377
LV-posterior wall, mm	16 (13; 17)	15 (13; 18)	15 (13; 18)	0.694
LV-EDD, mm	41 (38; 44)	41 (38; 46)	41 (38; 45)	0.697
LV-ESD, mm	31 (27; 34)	31 (28; 37)	31 (27; 35)	0.329
LV-mass/BSA, g/m²	143 (119; 164)	150 (124; 182)	147 (124; 180)	0.388
LV-EDV, mL	75 (57; 90)	78 (60; 96)	77 (60; 94)	0.146
LV-ESV, mL	36 (25; 46)	39 (32; 54)	38 (29; 50)	0.075
Ejection fraction, %	51 (44; 56)	49 (41; 55)	49 (43; 55)	0.120
MAPSE, mm	9 (7; 11)	8 (7; 10)	9 (7; 10)	0.138
TAPSE, mm	15(12; 20)	15 (11; 19)	15 (11; 19)	0.419
LV-GLS, %	−9.1 (−12; −7.1)	−8.1(−10; −6.4)	−8.4 (−10; −6.5)	0.013
RV-GLS, %	−15 (−20; −12)	−15 (−17; −10)	−15 (−19; −11)	0.201
Relative apical sparing, n (%)	23 (39)	52 (45)	75 (43)	0.432
LA-volume/BSA, mL/m²	44 (34; 55)	45 (32; 53)	44 (33; 54)	0.896
E-wave/A-wave	2.3 (1; 3.2)	2.6 (1.5; 3.4)	2.5 (1.4; 3.3)	0.191
E-wave/e‘-wave	16 (12; 20)	18 (13; 22)	17 (13; 22)	0.017
E-wave deceleration time, ms	158 (142; 206)	167 (135; 196)	164 (137; 206)	0.992
sPAP, mm Hg	40 (32; 46)	39 (32; 46)	39 (32; 46)	0.602
Pericardial effusion, n (%)	12 (20)	40 (35)	52 (30)	0.049

Results are given as number (percentage) or median (25th; 75th percentile).

BSA, body surface area; dFLC, difference of free light chains; EDD, end-diastolic diameter; EDV, end-diastolic volume; eGFR, estimated glomerular filtration rate; ESD, end-systolic diameter; ESV, end-systolic volume; GLS, global longitudinal strain; hsTnT, high sensitivity troponin T; LV, left ventricle; MAPSE, mitral annular plane systolic excursion; NT-proBNP, N-terminal pro-B-type natriuretic peptide; NYHA-FC, New York Heart Association functional class; RV, right ventricle; sPAP, systolic pulmonary artery pressure; TAPSE, tricuspid annular plane systolic excursion.

**Table 2:** Cardiac staging according to the MAYO 3b Score^18^

MAYO 3b stage	Survivors (n=59)	Non-survivors (n=115)	Total (n=174)	P value
I	–	–	–	0.016
II	25 (43)	27 (24)	52 (30)	
III	22 (37)	45 (39)	67 (39)	
IIIb	12 (20)	43 (37)	55 (31)	

None of the patients was in MAYO 3b stage I. Results are given as number (percentage).

In addition to cardiac involvement, at least one other organ was affected in 41% of the patients, and at least two other organs were affected in 10%. Soft tissue was the second most often involved organ system (24%), followed by gastrointestinal system and kidneys (17% and 6%, respectively). In our study, no difference was observed in the type or number of organs involved between survivors and non-survivors (online supplemental table 2). There was also no difference between both groups with regard to the underlying haematological disease. However, survivors had lower bone marrow plasma cell infiltration (online supplemental table 3).

Throughout the follow-up period, 94% of the patients received specific AL amyloidosis therapy. The therapy regimens are shown in online supplemental table 4. Bortezomib plus dexamethasone was the most frequently used treatment (53%). Other drugs commonly used in various combinations were cyclophosphamide, lenalinomide and melphalan. Bendamustine, doxorubicin and rituximab were ordered less often. One patient received stem cell transplantation (SCT). All others were classified as unsuitable for SCT due to advanced cardiac involvement. Ten patients remained untreated, all of whom belonged to the non-survivor group (five died before the therapy decision, three refused therapy, two were too old and in a significantly reduced general condition).

### Survival analysis

**Figure 2:**
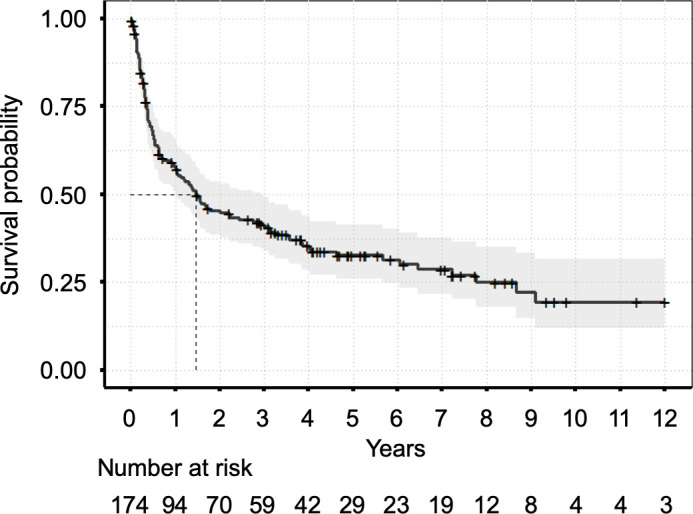
Kaplan-Meier curve displaying overall survival of the complete study cohort. Median survival time was 1.47 years.

The median OS time was 1.47 years (95% CI 1.04 to 2.92) or 17.6 moths (95% CI 12.5 to 35) figure 2. The median follow-up time was 5.16 years (95% CI 4.35 to 7.22) or 61.9 months (95% CI 52.2 to 86.6). The results of univariate Cox regression analysis and the final multivariable Cox regression model resulting from variable selection are presented in table 3. Male sex, blood pressure, NYHA-FC, low-voltage pattern (LVP), LV end-systolic volume (ESV), LV ejection fraction (EF), longitudinal ventricular function, pericardial effusion, NT-proBNP, hsTnT, dFLC and eGFR were associated with poor outcome, but only LV global longitudinal strain (HR 1.12; 95% CI 1.03 to 1.22; p=0.007), LV-ESV (HR 1.02; 95% CI 1.01 to 1.03; p=0.001), systolic pulmonary artery pressure (HR 0.98; 95% CI 0.96 to 0.99; p=0.027), NYHA-FC>II (HR 1.65; 95% CI 1.09 to 2.50; p=0.019), NT-proBNP (HR 1.57; 95% CI 1.17 to 2.11; p=0.003) and dFLC (HR 1.30; 95% CI 1.05 to 1.62; p=0.017) were independently predictive in the final Cox regression model.

**Table 3:** Univariate and multivariable Cox regression analysis

Parameter	Univariate Cox regression			Multivariable Cox regression		
	HR	95% CI	P value	HR	95% CI	P value
Male sex	1.641	1.096 to 2.456	0.016			
Age, years	1.007	0.987 to 1.026	0.513	1.009	0.988 to 1.030	0.407
Height, cm	1.014	0.996 to 1.032	0.137			
Weight, kg	1.007	0.995 to 1.019	0.242			
Systolic blood pressure, mm Hg	0.984	0.973 to 0.995	0.003			
Diastolic blood pressure, mmHg	0.977	0.960 to 0.995	0.011			
NYHA-FC>II	1.941	1.315 to 2.867	0.001	1.637	1.081 to 2.479	0.020
Light chain type lambda	0.747	0.456 to 1.224	0.247			
dFLC-log, mg/L	1.410	1.161 to 1.713	0.001	1.333	1.076 to 1.650	0.009
NT-proBNP-log, ng/L	1.989	1.554 to 2.546	<0.001	1.714	1.291 to 2.276	<0.001
hsTnT-log, pg/mL	1.563	1.188 to 2.058	0.001			
eGFR, mL/min/1.73 m^2^	0.985	0.977 to 0.994	0.001			
Heart rate/s	1.011	0.998 to 1.025	0.095			
Normal heart axis (reference)						
Left axis deviation	1.227	0.814 to 1.851	0.329			
Right axis deviation	1.117	0.651 to 1.916	0.687			
PQ interval, ms	0.998	0.992 to 1.003	0.385			
QRS interval, ms	1.004	0.994 to 1.013	0.428			
QTc interval, ms	0.998	0993 to 1.004	0.535			
Low-QRS-voltage pattern	1.842	1.269 to 2.672	0.001			
LV-septum, mm	1.053	0.995 to 1.115	0.074			
LV-posterior wall, mm	1.041	0.970 to 1.117	0.266			
LV-EDD, mm	1.000	0.972 to 1.029	0.993			
LV-ESD, mm	1.021	0.995 to 1.048	0.117			
LV-mass/BSA, g/m^2^	1.002	0.998 to 1.007	0.372			
LV-EDV, mL	1.004	0.997 to 1.011	0.240			
LV-ESV, mL	1.014	1.003 to 1.025	0.009	1.021	1.009 to 1.033	0.001
Ejection fraction, %	0.967	0.949 to 0.985	<0.001			
MAPSE	0.904	0.841 to 0.973	0.007			
TAPSE	0.950	0.913 to 0.989	0.012			
LV-GLS	1.151	1.073 to 1.235	<0.001	1.117	1.032 to 1.210	0.007
RV-GLS	1.036	1.002 to 1.072	0.040			
Relative apical sparing	1.332	0.921 to 1.926	0.127			
LA-Volumen/BSA, mL/m^2^	1.002	0.991 to 1.014	0.688			
E-wave/A-wave	1.192	1.018 to 1.395	0.029			
E-wave/e‘-wave	1.022	1.000 to 1.044	0.055			
E-wave Deceleration time, ms	0.999	0.995 to 1.002	0.462			
sPAP, mm Hg	0.995	0.978 to 1.013	0.582	0.975	0.955 to 0.995	0.016
Pericardial effusion	1.661	1.128 to 2.447	0.010			
Organs involved >1	0.913	0.629 to 1.326	0.632			
Organs involved >2	0.849	0.466 to 1.547	0.593			

BSA, body surface area; dFLC, difference of free light chains; EDD, end-diastolic diameter; EDV, end-diastolic volume; eGFR, estimated glomerular filtration rate; ESD, end-systolic diameter; ESV, end-systolic volume; GLS, global longitudinal strain; hsTnT, high sensitivity troponin T; LV, left ventricle; MAPSE, mitral annular plane systolic excursion; NT-proBNP, N-terminal pro-B-type natriuretic peptide; NYHA-FC, New York Heart Association functional class; RV, right ventricle; sPAP, systolic pulmonary artery pressure; TAPSE, tricuspid annular plane systolic excursion.

### Cut-off calculation and risk stratification

**Figure 3:**
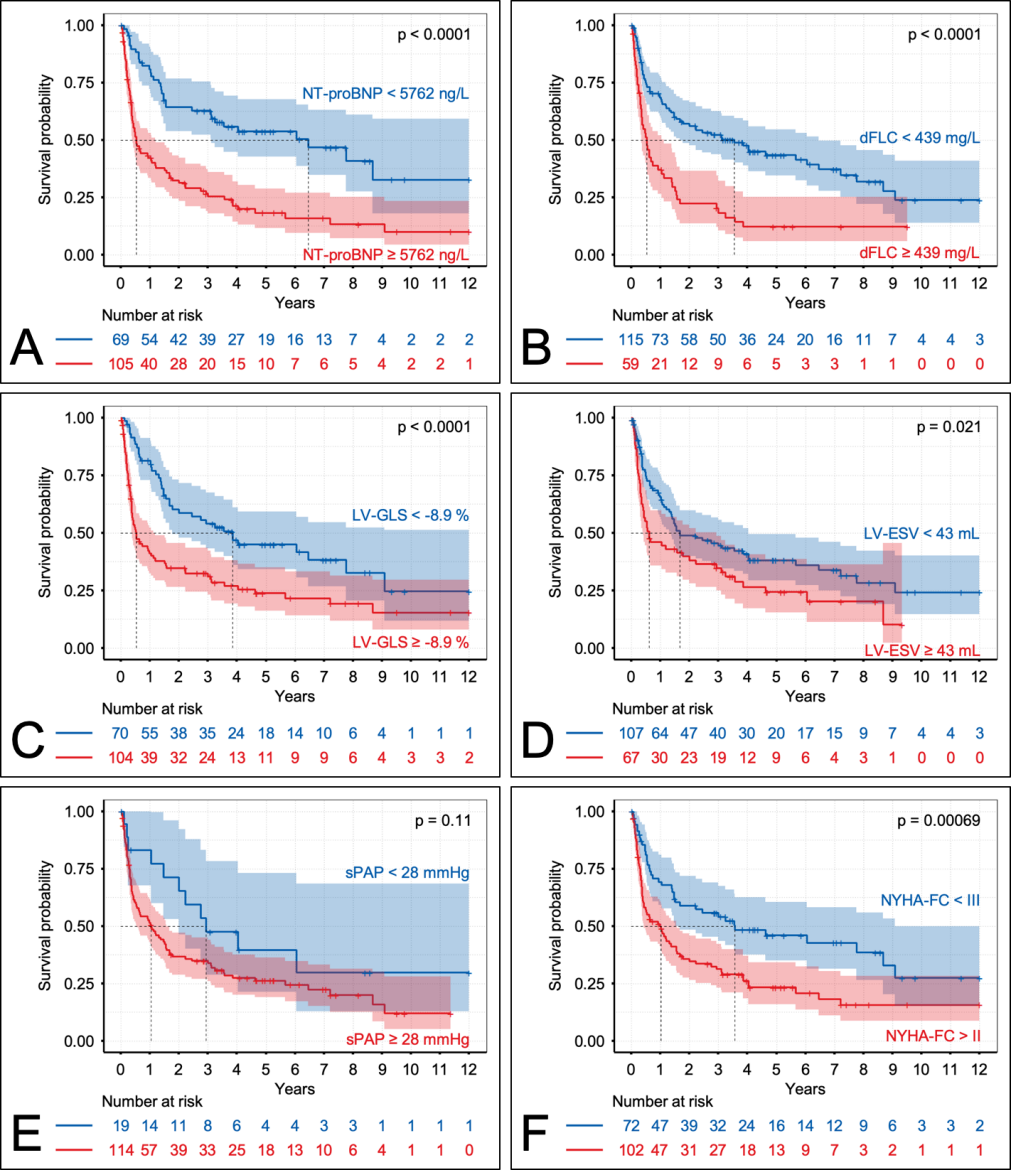
Kaplan-Meier curves displaying the survival probability according to parameters that were independent predictors in the multivariable Cox regression analysis. Stratification was done using individually calculated thresholds for each parameter. dFLC, difference of free light chains; ESV, end-systolic volume; LV-GLS, left ventricular-global longitudinal strain; NT-proBNP, N-terminal pro-brain natriuretic peptide; NYHA-FC, New York Heart Association functional class; sPAP, systolic pulmonary artery pressure.

**Figure 4:**
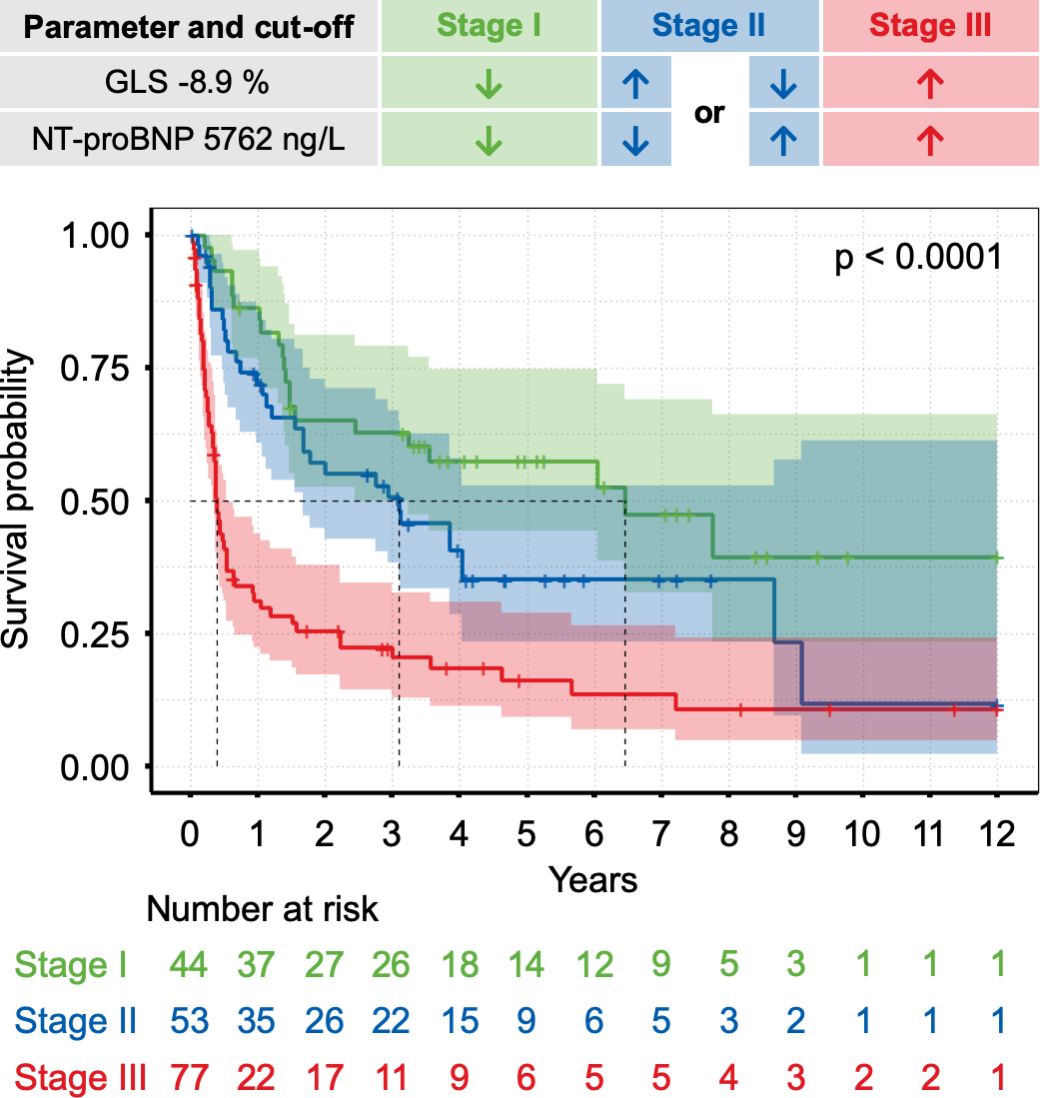
Risk stratification based on a scoring system incorporating N-terminal pro-B-type natriuretic peptide (NT-proBNP) and global longitudinal strain (GLS). The thresholds were 5762 ng/L for NT-proBNP and −8.9% for GLS. A combination of both parameters separated groups with lower (both parameters below the threshold)=stage I, intermediate (one parameter above the threshold)=stage II and higher mortality risk (both parameters above the threshold)=stage III.

For all continuous parameters that were independent predictors of mortality in the final Cox regression model, their individual optimal cut-off values were computed (table 4). Corresponding Kaplan-Meier curves using the identified thresholds of NT-proBNP (5762 ng/L), global longitudinal strain (−8.9%), LV-ESV (43 mL), systolic pulmonary artery pressure (28 mm Hg) and dFLC (439 mg/L) for stratification are presented in figure 3A–F. Based on the aforementioned cut-off values a staging system incorporating global longitudinal strain and NT-proBNP was developed. The resulting stratification of our study population into three patient cohorts with low (both parameters below the threshold), intermediate (one of the two parameters above the threshold) and high risk (both parameters above the threshold) is shown in figure 4.

**Table 4:** Cut-off values for risk stratification with corresponding 95% bootstrap percentile of continuous variables

Parameter	Optimal cut-off	2.5%	97.5%
GLS, %	−8.9	−10.2	−6.5
LV-ESV, mL	43	22	62
sPAP, mm Hg	28	28	53
NT-proBNP, ng/L	5762	4337	8840
dFLC, mg/L	439	220	537

dFLC, difference of free light chains; GLS, global longitudinal strain; LV-ESV, left ventricular end-systolic volume; NT-proBNP, N-terminal pro-B-type natriuretic peptide; sPAP, systolic pulmonary artery pressure.

## Discussion

This study aimed to investigate clinical, laboratory, electrocardiography and echocardiographic parameters for usefulness to predict outcome in AL amyloidosis with cardiac involvement proven by EMB.

Besides response to therapy, cardiac involvement is the main prognostic determinant in AL amyloidosis.^27^ Patients with cardiac involvement have a shorter survival than those without cardiac involvement, and mortality is even higher when congestive HF occurs.^28 29^ The search for prognostic markers therefore plays a crucial role, as they might aid in selecting therapeutic strategies, not only targeting the clonal disease but also regarding a timely and appropriate HF management.

Serum levels of NT-proBNP and cardiac troponins have emerged as important prognostic parameters and are therefore commonly used for risk stratification.^16 18^ In an unselected population of patients with AL amyloidosis the prognostic value of these biomarkers results from separating patients with a low (MAYO stage I) from those with a high probability of cardiac involvement (MAYO stage>I). Furthermore, they subdivide patients with suspected or proven cardiac involvement into a group with milder (MAYO stage II) and more advanced HF (MAYO stage III and IIIb). Beyond serological testing, functional cardiac imaging, particularly strain analysis, plays a fundamental role in risk stratification and prognostication in AL amyloidosis.^30^ However, the aforementioned parameters must be interpreted with caution because increased levels of biomarkers and pathological imaging findings can also be found in the context of other diseases and do not necessarily confirm or reflect cardiac involvement in AL amyloidosis.

The gold standard for the diagnosis of cardiac involvement is EMB.^31 32^ Due to its invasiveness, EMB is not common in most studies investigating survival criteria in AL amyloidosis in general^23 33 34^ and few studies have investigated the prognosis of patients with cardiac involvement proven by EMB in particular. These studies identified sex, Karnofsky index, diastolic blood pressure, NYHA-FC, NT-proBNP, GFR, mineralocorticoid receptor antagonist use, lack of β-blocker use, E-wave deceleration time, LV-EF, LV mass, LV wall thickness, LVP, intraventricular conduction delay, amyloid load, response to chemotherapy and SCT as markers associated with survival.^22 35–37^ Only LV-EF, LVP,^35^ NYHA-FC,^36^ NT-proBNP, GFR, response to chemotherapy and amyloid load^37^ were independent predictors of mortality in the multivariable model. However, cardiac deformation assessed by strain imaging had not been investigated for its prognostic value in patients with AL amyloidosis and EMB-proven cardiac involvement so far.

To close this knowledge gap, we screened the database of the amyloidosis centre at Heidelberg University Hospital, one of the largest of its kind worldwide. Among 1628 patients who had their initial visit from July 2006 to December 2017, we identified 216 patients with EMB proven cardiac involvement. To eliminate the effect of chemotherapy and to reduce the influence of comorbidities on our analysis, only therapy-naïve patients without a history of significant cardiac or renal disease at the time of EMB were further investigated. At the end of the screening process 174 patients met the criteria for inclusion in this study.

The median OS of the complete cohort was 1.47 years (95% CI 1.04 to 2.92 years) which is comparable with the literature on patients with AL amyloidosis.^6 28 29^ One hundred and fifteen patients (66%) had died after the 12 years of the investigation. The survival probability at 10 years from the time of first contact with the amyloidosis centre was 0.195 (95% CI 0.120 to 0.316).

Comparing survivors with non-survivors male sex, blood pressure, NYHA-FC, LVP, LV- ESV, LV-EF, longitudinal ventricular function, pericardial effusion, NT-proBNP, hsTNT, dFLC and eGFR were associated with poor outcome but only NYHA-FC >II, LV global longitudinal strain, LV-ESV, systolic pulmonary artery pressure, NT-proBNP and dFLC were independently predictive in multivariable analysis. Interestingly, from the time of EMB proven cardiac involvement, neither the serum level of cardiac hsTNT, as an indicator of myocardial damage and integral component of current amyloidosis staging systems, nor LV-EF, as a standard measure of cardiac systolic function and crucial criterion for HF classification, had independent prognostic significance in our study cohort. However, this does not mean that both parameters have no predictive value at all. Rather our variable selection was subject to some restrictions and the circumstances of missing values might have introduced additional uncertainty. Due to the number of predictors in relation to the effective sample size for multivariable Cox regression, the better approach of backward elimination was not feasible and so forward selection was performed for variable selection.

New treatment options have improved the survival of patients with AL amyloidosis over the past few decades.^20^ A basic requirement before starting therapy is optimal risk stratification. However, only patients in MAYO stage I had significant survival improvement over time, while the survival improvement in MAYO stage II and III/IIIb was marginal or nil, respectively.^38^ Risk stratification tools in patients with both, AL amyloidosis and cardiac involvement, should therefore provide a better predictive power. A first step to improve the original MAYO score was to implement a more appropriate cut-off level for NT-proBNP to identify a very poor-prognosis subgroup of patients in MAYO stage III.^18^ From the cardiologist’s point of view additional options exist, that could supplement a prognostic evaluation based purely on blood tests. In this context, cardiac imaging holds great potential as it provides direct and objective structural and functional read-outs. A focused analysis of myocardial performance may be superior to clinical classification systems like NYHA-FC, the assessment of which may vary substantially, or biomarkers such as dFLC that primarily reflects haematological disease burden.^17^

Global longitudinal strain could be a promising candidate to optimise the risk stratification of cardiac AL amyloidosis. Compared with other echocardiographic parameters that were independently predictive in our study, global longitudinal strain has lower intraobserver and interobserver variability than LV-ESV^39 40^ and, unlike systolic pulmonary artery pressure, should be measurable in most patients. Furthermore, recent studies have demonstrated that strain imaging can predict survival and therapy response in AL amyloidosis in general^41–45^ while our data further suggest that global longitudinal strain in combination with NT-proBNP may offer a useful survival risk stratification for AL amyloidosis patients with cardiac involvement in particular. However, a prognostic benefit for patients with cardiac AL amyloidosis needs to be prospectively validated and tested against other accepted staging systems in the field.

The inclusion criteria of our study resulted in a highly selected patient cohort especially since some comorbidities such as atrial fibrillation might have been directly related to AL amyloidosis. Furthermore, the rates of atrial fibrillation, additional kidney amyloidosis and the use of SCT were very low compared with a general population with AL amyloidosis. Therefore, our findings are not transferable to all AL amyloidosis patients, especially to those without cardiac involvement.

### Limitations

Although the amyloidosis centre at Heidelberg University Hospital has a nationwide catchment area, this is a single-centre study. The retrospective nature of our study and the requirement for EMB proven cardiac involvement may contribute to a selection bias. We minimised any bias by including all patients with AL amyloidosis and cardiac involvement during the above-mentioned time interval. Nevertheless, we want to point out that the cohort examined in this study is not representative of the total population of patients diagnosed with AL amyloidosis. This is reflected, among other things, in a low rate of concomitant renal involvement or atrial fibrillation.

The onset and therefore the true duration of AL amyloidosis are difficult to assess because patients often show no clinical signs even at the beginning of cardiac involvement. The fact that all patients in our study underwent EMB indicates a high suspicion for an advanced stage of the disease in our cohort.

While the multivariable Cox regression analysis identified predictors for mortality, other important indicators may have been missed. Additionally, the variable selection might have suffered from the uncertainty due to the multiple imputation.

## Conclusion

Among all patients with AL amyloidosis those with cardiac involvement represent a high-risk population with limited therapy options and poor prognosis. However, the present study suggests, that a relevant number of patients with cardiac AL amyloidosis can survive many years after the initial diagnosis. Therefore, accurate risk stratification is necessary to identify cardiac amyloidosis patients with favourable prognosis. Incorporation of modern imaging techniques into existing or newly developed scoring systems is a promising option that might enable the implementation of risk-adapted therapeutic strategies.

## Data Availability

Data are available on reasonable request.
