# Supplementary material for: Prognosis of light chain amyloidosis: a multivariable analysis for survival prediction in patients with cardiac involvement proven by endomyocardial biopsy
Source: Open Heart. 2023 Jul 19;10(2):e002310. doi: 10.1136/openhrt-2023-002310 (PMC10357742; doi:10.1136/openhrt-2023-002310)
Supplement: Supplementary data [file openhrt-2023-002310supp001.pdf]

Parameter	Survivors (n = 94)	Nonsurvivors (n = 71)	p value
**Basic and clinical data**			
Male sex, n (%)	56 (60)	54 (76)	0.026
Age, years	63 [56; 69]	61 [55; 70]	0.867
Hight, cm	172 [165; 182]	177 [170; 81]	0.195
Weight, kg	75 [64; 84]	78 [70: 87]	0.094
Systolic blood pressure, mmHg	110 [100; 125]	105 [100; 110]	<0.001
Diastolic blood pressure, mmHg	75 [70; 80]	70 [65; 75]	0.001
NYHA-FC > II, n (%)	47 (50)	50 (70)	0.008
**Clinical chemistry**			
Light chain type Lambda, n (%)	83 (88)	57 (80)	0.155
dFLC, mg/L	244 [135; 421]	439 [238; 841]	<0.001
NT-proBNP, ng/L	5030 [2576; 8132]	9783 [6344; 17927]	<0.001
hsTnT, pg/mL	59 [40; 98]	91 [48; 157]	0.003
eGFR, ml/min/1.73 m^2	84 [71; 99]	68 [57;87]	<0.001
**Electrocardiogram**			
Sinus rhythm, n (%)	94 (100)	71 (100)	-
Heart rate, s^{-1}	80 [68; 90]	83 [75; 91]	0.122
Heart axis			0.264
- Normal heart axis, n (%)	37 (39)	21 (30)	
- Left axis deviation, n (%)	39 (41)	38 (54)	
- Right axis deviation, n (%)	18 (19)	11 (16)	
PQ interval, ms	180 [164; 213]	179 [154; 210]	0.294
QRS interval, ms	96 [86; 111]	102 [96; 112]	0.041
QTc interval, ms	437 [418; 457]	441 [422; 455]	0.612
Low-QRS-voltage pattern, n (%)	27 (29)	38 (55)	<0.001
**Echocardiogram**			
LV-Septum, mm	16 [15; 20]	19 [16; 21]	0.007
LV-posterior wall, mm	15 [13; 17]	16 [14; 18]	0.092
LV-EDD, mm	42 [38; 45]	40 [38; 45]	0.625
LV-ESD, mm	30 [27; 34]	31 [29; 38]	0.035
LV-mass/BSA, g/m²	143 [120; 168]	153 [135; 182]	0.172
LV-EDV, mL	76 [60; 93]	80 [60; 99]	0.306
LV-ESV, mL	36 [26; 47]	43 [32; 56]	0.009
Ejection fraction, %	52 [45; 59]	47 [40; 53]	<0.001
MAPSE, mm	9 [8; 11]	8 [7; 9]	<0.001
TAPSE, mm	15 [12; 20]	14 [10; 18]	0.008
LV-GLS, %	-9.6 [-12.0; -7.5]	-7.3 [-8.5; -6.0]	0.001
RV-GLS, %	-15.0 [-20.0; -12.0]	-13.0 [-16.0; -9.8]	0.004
Relative apical sparing, n (%)	36 (38)	36 (51)	0.112
LA-Volume/BSA, mL/m²	43 [32; 54]	46 [35; 55]	0.312
E-wave/A-wave	2.3 [1.1; 3.2]	2,6 [1.8; 3.4]	0.076
E-wave/e‘-wave	17 [12; 22]	18 [13; 23]	0.112
E-wave Deceleration time, ms	168 [141; 210]	161 [133; 192]	0.121
sPAP, mmHg	38 [32; 46]	41 [32; 46]	0.908
Pericardial effusion, n (%)	25 (27)	25 (35)	0.233

Supplement Table 1: Comparison of survivors and nonsurvivors with 1 year of follow-up (9 censored patients were excluded).

BSA, body surface area; dFLC, difference between involved and uninvolved free light chains; EDD, end-diastolic diameter; EDV, end-diastolic volume; eGFR, estimated glomerular filtration rate; ESD, end-systolic diameter; ESV, end-systolic volume; GLS, global longitudinal strain; hsTnT, high sensitivity troponin T; LV, left ventricle; MAPSE, mitral annular plane systolic excursion; NT-proBNP, N-terminal pro-B-type natriuretic peptide; NYHA-FC, New York Heart Association functional class; RV, right ventricle; sPAP, systolic pulmonary artery pressure; TAPSE, tricuspid annular plane systolic excursion

Results are given as number (percentage) or median [25th; 75th percentile]

Organ	Survivors (n = 59)	Nonsurvivors (n = 115)	Total (n = 174)	p value
Heart, n (%)	59 (100)	115 (100)	174 (100)	-
Soft tissue, n (%)	19 (32)	23 (20)	42 (24)	0.075
Gastro intestinal tract, n (%)	7 (12)	22 (19)	29 (17)	0.223
Kidney, n (%)	4 (7)	6 (5)	10 (6)	0.675
Liver / Gall bladder, n (%)	1 (2)	2 (2)	3 (2)	n.a.
Lung / Pleura, n (%)	-	1 (1)	1 (1)	n.a.
Skin, n (%)	-	1 (1)	1 (1)	n.a.
Total organ involvement	1 [1; 2]	1 [1; 2]	1 [1; 2]	0.997
Organs involved > 1, n (%)	24 (41)	47 (41)	71 (41)	0.981
Organs involved > 2, n (%)	6 (10)	12 (10)	18 (10)	0.957

Supplement Table 2: Organ involvement
Results are given as number (percentage); n.a., not applicable due to low number of cases

Hematological diagnosis and bone marrow plasma cell infiltration	Survivors (n = 59)	Nonsurvivors (n = 115)	Total (n = 174)	p value
Monoclonal gammopathy, n (%)	82 (74)	44 (75)	126 (74)	0.921
Multiple myeloma, n (%)	12 (11)	3 (5)	15 (9)	0.210
Smoldering myeloma, n (%)	16 (14)	12 (20)	28 (16)	0.321
Waldenstrom's disease, n (%)	1 (1)	-	1 (1)	n.a.
Bone marrow plasma cell infiltration, %	12 [8; 20]	10 [8;14]	12 [8; 19]	0.024

Supplement Table 3: Hematological data
Results are given as number (percentage); n.a., not applicable due to low number of cases

Therapy schema	Survivors (n = 59)	Nonsurvivors (n = 115)	Total (n = 174)	p value
Bor-Dex, n (%)	34 (58)	58 (50)	92 (53)	n.a.
M-Dex, n (%)	8 (14)	24 (21)	32 (18)	
Cy-Bor-Dex, n (%)	10 (17)	9 (8)	19 (11)	
Lenalindomide-M-Dex, n (%)	4 (7)	10 (9)	14 (8)	
Other, n (%)	3 (5)	4 (3)	7 (4)	
No specific Therapy, n (%)	-	10 (9)	10 (6)	

Supplement Table 4: Therapy schemata
Bor: bortezomib; Cy: cyclophosphamide; Dex: dexamethasone; M: melphalan; Other includes bendamustin, doxorubicin, rituximab and 1 patient with stem cell transplantation
Results are given as number (percentage); n.a., not applicable due to low number of cases
